# The impact of different left ventricular geometric patterns on right ventricular deformation and function in the elderly with hypertension: A two-dimensional speckle tracking and three-dimensional echocardiographic study

**DOI:** 10.3389/fcvm.2022.929792

**Published:** 2022-10-18

**Authors:** Jiping Xue, Xiaoyan Kang, Qin Qin, Junwang Miao, Shuai Li, Chunsong Kang

**Affiliations:** Department of Ultrasonography, Shanxi Bethune Hospital, Shanxi Academy of Medical Sciences, Tongji Shanxi Hospital, The Third Hospital of Shanxi Medical University, Taiyuan, China

**Keywords:** hypertension, left ventricular geometric pattern, right ventricle, two-dimensional speckle tracking, three-dimensional echocardiography

## Abstract

**Objective:**

This study aimed to evaluate the impact of different left ventricular geometric patterns on right ventricular deformation and function in the elderly with essential hypertension *via* two-dimensional speckle tracking and three-dimensional echocardiography.

**Methods:**

A total of 248 elderly people with essential hypertension were divided into four groups based on the left ventricular mass index (LVMI) and relative wall thickness (RWT): the normal geometric, concentric remodeling, eccentric hypertrophy, and concentric hypertrophy groups. Moreover, 71 participants were recruited as the control group. These participants were examined by two-dimensional speckle tracking and three-dimensional echocardiography to obtain the right ventricular strain parameters, three-dimensional volume, and function parameters.

**Results:**

The right ventricular strain parameters decreased gradually from the normal geometric group to the concentric hypertrophy group (*P* < 0.05), and the strain parameters in the concentric remodeling, eccentric hypertrophy, and concentric hypertrophy groups were lower than those in the control and normal geometric groups (*P* < 0.05). The right ventricular three-dimensional echocardiographic parameters only changed in the eccentric hypertrophy group (*P* < 0.05) and the concentric hypertrophy group (*P* < 0.05) in the form of an increase in volume and a decrease in function. Multivariate linear regression analysis showed that the right ventricular free wall longitudinal strain was independently associated with the systolic blood pressure (SBP), LVMI, and RWT (*P* < 0.05) and was primarily affected by the LVMI (normalized β = 0.637, *P* < 0.05).

**Conclusion:**

The systolic function of the right ventricular myocardium declined in the elderly with essential hypertension due to impaired myocardial mechanics. The right ventricular strain parameters could indicate mechanical damage in the concentric remodeling group earlier than the right ventricular three-dimensional volume and function parameters. The right ventricular free wall longitudinal strain was primarily subject to the LVMI.

## Introduction

Hypertension is a common chronic disease leading to end organ damage and is a factor in multiple cardiovascular complications, including stroke, coronary heart disease, and heart failure. The number of elderly patients with hypertension is increasing annually, with relatively high morbidity, disability, and mortality rates (1). Long-term hypertension leads to compensatory cardiomyocyte hyperplasia and hypertrophy, resulting in increased myocardial weight, enlargement of the ventricular chamber, and a change in the left ventricular geometric pattern. Based on the left ventricular mass index (LVMI) and relative wall thickness (RWT), Ganau et al. (2) classified left ventricular geometric patterns into normal geometric, concentric remodeling, eccentric hypertrophy, and concentric hypertrophy patterns. The left ventricular geometric pattern has independent prognostic significance for the morbidity and mortality of cardiovascular diseases (3). Garg et al. (4) have shown that the incidence of heart failure or cardiovascular death is 2.0% in patients with normal geometric and concentric remodeling and 2.8% in patients with eccentric hypertrophy, rising sharply to 11.8% in patients with concentric hypertrophy.

The left and right ventricles are anatomically enclosed within the same pericardial cavity (sharing the interventricular septum). They are wound by the same helical ventricular myocardial band; thus, they affect each other's structure and function (5). Studies have shown that hypertension may lead to changes in the right ventricular structure and systolic function. A growing body of evidence demonstrates that right ventricular function is the key factor for cardiovascular disease prognosis (6). Early diagnosis of right ventricular morphology and function changes may help prevent or delay heart failure and cardiovascular death (7). However, the extent of right ventricular morphology and function change in patients with hypertension with different left ventricular geometric patterns is still disputed. Tadic et al. (8) have shown that the right ventricular volume of patients with left ventricular hypertrophy, especially concentric hypertrophy, is significantly larger than that of patients without left ventricular hypertrophy, the right ventricular ejection fraction (RVEF) decreases, and the tricuspid annular plane systolic excursion (TAPSE) only decreases in patients with concentric hypertrophy. However, Karaye et al. (9) have stated that increased right ventricular volume and decreased TAPSE are only observed in patients with eccentric hypertrophy and that conventional echocardiographic parameters are not sensitive to right ventricular function damage in patients with early left ventricular remodeling. Studies have shown that two-dimensional speckle tracking imaging can quantify myocardial function, measuring the right ventricular longitudinal strain as an early sensitive index to evaluate right ventricular systolic function, which can also function as an independent risk factor for the prognosis of multiple cardiovascular diseases (10–12). Therefore, in this study, two-dimensional speckle tracking was used in combination with three-dimensional echocardiography to evaluate the right ventricular deformation and function of elderly people with hypertension with different left ventricular geometric patterns, thereby detecting early mechanical damage to the right ventricle and providing a basis for early intervention and prognosis.

## Materials and methods

### Subjects

A total of 285 elderly patients diagnosed with essential hypertension in Shanxi Bethune Hospital from January to June 2017 were recruited for this study; 37 cases were excluded due to low-quality images, and the remaining 248 cases were included, comprising 122 males and 126 females aged 65–79 (68.46 ± 2.73) years. Another 71 elderly people with a corresponding age and gender distribution and without cardiovascular disease were recruited as the control group, comprising 32 males and 39 females aged 65–72 (68.34 ± 2.53) years. This study was approved by the medical ethics committee of the hospital (YXLL-2020-045). All subjects signed the informed consent form.

The inclusion criteria were as follows: patients aged 65–79 years; patients whose blood pressure (measured three times across different days) showed a systolic blood pressure (SBP) ≥ 140 mmHg and/or diastolic blood pressure (DBP) ≥ 90 mmHg without the use of antihypertensive drugs, and patients who received medical treatment to control blood pressure and were diagnosed as elderly with hypertension despite their blood pressure being less than 140/90 mmHg.

The exclusion criteria were as follows: elderly patients suffering from secondary hypertension, pulmonary hypertension, history of stroke, dilated or hypertrophic cardiomyopathy, rheumatic heart disease, congenital heart disease, severe valvular heart disease, acute myocardial infarction or unstable angina pectoris, diabetes, and severe hepatic or renal diseases.

### Instruments

The GE Vivid E9 color Doppler ultrasound (GE Healthcare, Horten, Norway), M5S transducer (1.5–4.5 MHz), 4V real-time three-dimensional cardiac volume transducer (1.5–4.5 MHz), and EchoPAC analysis software (version 203) were used.

### Methods

#### Clinical data acquisition

The age, gender, heart rate, height, weight, and hypertension duration of the subjects were measured and recorded to calculate the body mass index (BMI) and body surface area (BSA) as follows: BMI = height(kg)/weight(m)^2^, and BSA = 0.0061 × height + 0.0128 × weight – 0.1529. For the blood pressure measurement, each subject was allowed to sit down and wait for 15 min in the resting state; then, sphygmomanometry was performed to take three blood pressure readings at an interval of 3 min from the right upper arm, which was lifted to the level of the heart. Finally, the average of the three measures was calculated, and the SBP and DBP were recorded.

#### Image acquisition

Echocardiography was performed on every subject. Each subject was instructed to take a left lateral position and breathe normally, and the echocardiogram machine was connected synchronously. Then, M-mode echocardiography was performed on the long-axis section of the left ventricle to obtain the interventricular septum end-diastolic thickness (IVSTd), left ventricular posterior wall end-diastolic thickness (LVPWTd), left ventricular end-diastolic diameter (LVEDD), and left ventricular end-systolic diameter (LVESD). The left ventricular ejection fraction (LVEF) was calculated using the biplane Simpson method from the apical four-chamber section. The above measurements were averaged across three cardiac cycles. The LVM was calculated with the formula specified in the American Society of Echocardiography (ASE) guidelines as follows: LVM = 0.8 × 1.04 × [(IVSTd + LVPWTd + LVEDD)^3^ – LVEDD^3^] + 0.6 g. The LVMI was corrected by the BSA as follows: LVMI = LVM/BSA. Finally, the RWT was calculated as follows: (2 × LVPWTd)/LVEDD. The left ventricular geometric patterns were divided into four categories by the LVMI and RWT: normal geometric (normal LVMI and RWT), concentric remodeling (normal LVMI and RWT exceeding cutoff value), eccentric hypertrophy (LVMI exceeding cutoff value and normal RWT), and concentric hypertrophy (LVMI and RWT exceeding cutoff values).

According to the ASE guidelines (2015) (13), the cutoff values for LVMI are 95 g/m^2^ (female) and 115 g/m^2^ (male), and that for RWT is 0.42 (irrespective of gender).

In addition, each subject was instructed to hold their breath after exhaling, and two-dimensional gray-scale dynamic images (frame frequency: 60–120/s) were continuously collected from the apical four-chamber section throughout at least three cardiac cycles. These images were stored and analyzed offline to obtain the right ventricular strain parameters. Next, the 4V transducer was enabled, and the four-dimensional full volume function key was activated. The subject was instructed to hold their breath after exhaling, and full-volume images (frame frequency adjusted to 40% of the subject's heart rate) were continuously collected from the apical four-chamber section throughout 4–6 cardiac cycles. These images were stored and analyzed offline to obtain the right ventricular three-dimensional volume and function parameters.

#### Image analysis

The image analysis was conducted in the EchoPAC workstation by importing each subject's two-dimensional gray-scale dynamic images, entering the Q-Analysis strain analysis program, and clicking “2D Strain” to trace when the right ventricular endocardial surface was clear. The software automatically generated the region of interest; the width and position were manually adjusted to adapt to the thickness of the myocardial wall and exclude the pericardium. Then, “Approve” was clicked to obtain the right ventricular free wall longitudinal strain peak and right interventricular septum longitudinal strain. Afterward, the right ventricular full-volume image of the subject was imported, and “4D Auto RVQ” was clicked to manually trace the right ventricular endocardium. The software automatically obtained the right ventricular end-diastolic volume (RVEDV), right ventricular end-systolic volume (RVESV), RVEF, TAPSE, and right ventricular fractional area change.

### Reproducibility studies

Twenty subjects were randomly selected for imaging. Two experienced doctors were assigned to analyze the images obtained in the abovementioned manner and measure and record the right ventricular free wall longitudinal strain and right interventricular septum longitudinal strain, thus carrying out the interobserver reproducibility study. Then, either of the doctors analyzed these data again after a week to carry out the intra-observer reproducibility study.

### Statistical analysis

Statistical software, SPSS 24.0, was used to analyze these data. The Kolmogorov–Smirnov test was used with these measurement data for the normality test. The normally distributed data were expressed as the mean ± standard deviation. The data among multiple groups were compared with a one-way analysis of variance (ANOVA), and the least significant difference (LSD)-*t-*test was used for multiple comparisons. Cases expressed the data, and the χ^2^ test was used for comparisons among groups. Univariate and multivariate linear regression analyses were applied to the correlation between right ventricular free wall longitudinal strain (RVFWLS) and clinical and echocardiographic parameters. A *P*-value < 0.05 indicated that the difference was statistically significant.

## Results

### Comparison of general data

Compared with the control group, the SBP and DBP were increased in the hypertension groups (with varied geometric patterns), and the differences were statistically significant (*P* < 0.05). Compared with the normal geometric group, the SBP, DBP, and hypertension duration were increased in the eccentric hypertrophy and concentric hypertrophy groups, and the differences were statistically significant (*P* < 0.05). There was no significant difference in gender, age, BMI, or heart rate between the control and hypertension groups (*P* > 0.05; Table 1).

**Table 1:** Comparison of general data ($\bar{x}$ ± s).

**Group**	***n***	**Gender**	**Age** ** (years)**	**SBP** ** (mmHg)**	**DBP** ** (mmHg)**	**BMI** ** (kg/m^2^)**	**HR** ** (Bpm)**	**Hypertension duration** ** (years)**
		**(n, male/female)**						
Control group	71	32/39	68.34 ± 2.53	126.21± 5.00	76.57 ± 3.22	23.88 ± 0.54	75.52 ± 5.55	0
NG	88	43/45	68.47 ± 2.78	150.08 ± 5.02^a^	80.26 ± 3.81^a^	24.05 ± 0.61	75.53 ± 5.83	11.90 ± 3.57
CR	70	36/34	68.18 ± 2.68	150.68 ± 4.10^a^	81.11 ± 3.36^a^	23.97 ± 0.70	75.34 ± 5.77	12.80 ± 4.16
EH	37	18/19	69.25 ± 2.55	152.44 ±4.36^ab^	82.11 ±3.50^ab^	23.90 ± 0.60	75.62 ± 5.48	13.72 ± 3.23^b^
CH	53	25/28	68.27 ± 2.82	151.70 ±4.59^ab^	82.22 ± 3.60^ab^	23.97 ± 0.50	76.58 ± 4.98	13.99 ± 4.20^b^
*F*/χ^2^ value		0.38	1.07	387.42	27.15	0.81	0.44	3.98
*P* value	-	0.54	0.36	< 0.001	< 0.001	0.52	0.78	0.009

^a^Compared with the control group, *p* < 0.05; ^b^Compared with the normal geometric group, *p* < 0.05. BMI, body mass index; SBP, systolic blood pressure; DBP, diastolic blood pressure; HR, heart rate; NG, normal geometric; CR, concentric remodeling; EH, eccentric hypertrophy; CH, concentric hypertrophy; and 1mmHg = 0.133kPa.

### Comparison among groups in terms of conventional left ventricular echocardiographic parameters

Compared with the other four groups, the IVSTd, LVPWTd, LVMI, and RWT were increased in the concentric hypertrophy group; compared with the control and normal geometric groups, the LVEF was decreased in the concentric hypertrophy group.

Compared with the other four groups, the LVEDD and LVESD were increased in the eccentric hypertrophy group; compared with the control, normal geometric, and concentric remodeling groups, the LVMI was increased in the eccentric hypertrophy group; the differences were statistically significant (*P* < 0.05).

Compared with control and normal geometric groups, the IVSTd, LVPWTd, LVMI, and RWT were increased in the concentric remodeling group, and the differences were statistically significant (*P* < 0.05; Table 2).

**Table 2:** Comparison among groups in terms of left ventricular echocardiographic parameters ($\bar{x}$ ± s).

**Groups**	**n**	**IVSTd** ** (mm)**	**LVPWTd** ** (mm)**	**LVEDD** ** (mm)**	**LVESD** ** (mm)**	**LVEF** ** (%)**	**LVMI** ** (g/m^2^)**	**RWT**
Control group	71	9.16 ± 0.95	9.23 ± 0.72	47.20 ± 2.47	29.08 ± 2.15	65.03 ± 2.23	91.91 ± 5.95	0.38 ± 0.01
NG	88	9.16 ± 0.79	9.41 ± 0.77	47.28 ± 2.50	29.66 ± 2.40	65.20 ± 1.77	92.31 ± 5.21	0.38 ± 0.02
CR	70	9.84 ± 0.96^ab^	9.98 ± 0.85^ab^	47.09 ± 2.21	29.32 ± 2.83	64.66 ± 1.84	96.01 ± 4.56^ab^	0.43 ± 0.02^ab^
EH	37	10.17 ± 0.90^ab^	10.19 ± 0.85^ab^	51.30 ± 2.02^abc^	32.36 ± 2.31^abc^	64.76 ± 2.24	118.28 ± 4.94^abc^	0.38 ± 0.02^c^
CH	53	11.13 ± 1.07^abcd^	11.26 ± 0.73^abcd^	49.72 ± 2.31^abcd^	30.77 ± 2.34^abcd^	64.02 ± 2.20^ab^	123.97 ± 5.46^abcd^	0.47 ± 0.02^abcd^
F value		47.37	62.66	32.02	14.33	3.12	479.47	269.45
*P* value		< 0.001	< 0.001	< 0.001	< 0.001	0.015	< 0.001	< 0.001

^a^Compared with the control group, *p* < 0.05; ^b^Compared with the normal geometric group, *P* < 0.05; ^c^Compared with the concentric remodeling group, *P* < 0.05; ^d^Compared with the eccentric hypertrophy group, *p* < 0.05. IVSTd, interventricular septum end-diastolic thickness; LVPWTd, left ventricular posterior wall end-diastolic thickness; LVEDD, left ventricular end-diastolic diameter; LVESD, left ventricular end-systolic diameter; LVMI, left ventricular mass index; RWT, relative wall thickness; and LVEF, left ventricular ejection fraction.

### Comparison among groups in terms of right ventricular three-dimensional echocardiographic parameters

Compared with the other three groups, the RVEDV and RVESV were increased in the eccentric hypertrophy and concentric hypertrophy groups. Compared with the control and normal geometric groups, the RVEF and FAC were decreased in eccentric hypertrophy and concentric hypertrophy; the differences were statistically significant (*P* < 0.05). The TAPSE of the concentric hypertrophy group was lower than that of the control and normal geometric groups, and the difference was statistically significant (*P* < 0.05; Table 3).

**Table 3:** Comparison among groups in terms of right ventricular echocardiographic parameters ($\bar{x}$ ± s).

**Groups**	***n***	**RVEDV** ** (ml)**	**RVESV** ** (ml)**	**RVEF** ** (%)**	**TAPSE** ** (mm)**	**FAC** ** (%)**	**Right interventricular septum longitudinal strain** ** (%)**	**Right ventricular free wall longitudinal strain** ** (%)**
Control group	71	86.79 ± 3.84	36.75 ± 3.31	57.33 ± 3.80	24.02 ± 1.74	53.55 ± 2.53	−22.53 ± 0.82	−29.81 ± 0.81
NG	88	87.80 ± 4.50	36.91 ± 3.72	56.72 ± 4.79	24.06 ± 1.94	53.69 ± 2.46	−22.57 ± 0.74	−29.68 ± 0.90
CR	70	88.11 ± 4.13	37.80 ± 3.21	56.16 ± 4.99	23.50 ± 2.05	53.21 ± 2.32	−21.72 ± 0.79^ab^	−28.30 ± 0.89^ab^
EH	37	92.25 ± 3.79^abc^	41.65 ± 2.56^abc^	54.67 ± 4.97^ab^	23.59 ± 1.47	52.46 ± 1.77^ab^	−20.25 ± 0.85^abc^	−27.01 ± 0.82^abc^
CH	53	92.75 ± 4.91^abc^	43.03 ± 2.85^abc^	54.69 ± 4.27^ab^	23.23 ± 1.69^ab^	52.43 ± 2.08^ab^	−19.27 ± 0.63^abcd^	−26.56 ± 0.73^abcd^
F value		22.68	45.09	3.83	2.47	3.74	212.30	184.05
*P* value		< 0.001	< 0.001	0.005	0.04	0.005	< 0.001	< 0.001

^a^Compared with the control group, p < 0.05; ^b^Compared with the normal geometric group, *P* < 0.05; ^c^Compared with the concentric remodeling group, *P* < 0.05; ^d^Compared with the eccentric hypertrophy group, *p* < 0.05. RVEDV, right ventricular end-diastolic volume; RVESV, right ventricular end-systolic volume; RVEF, right ventricular ejection fraction; TAPSE, tricuspid annular plane systolic excursion; and FAC, right ventricular fractional area change.

### Comparison among groups in terms of right ventricular strain parameters

**Figure 1:**
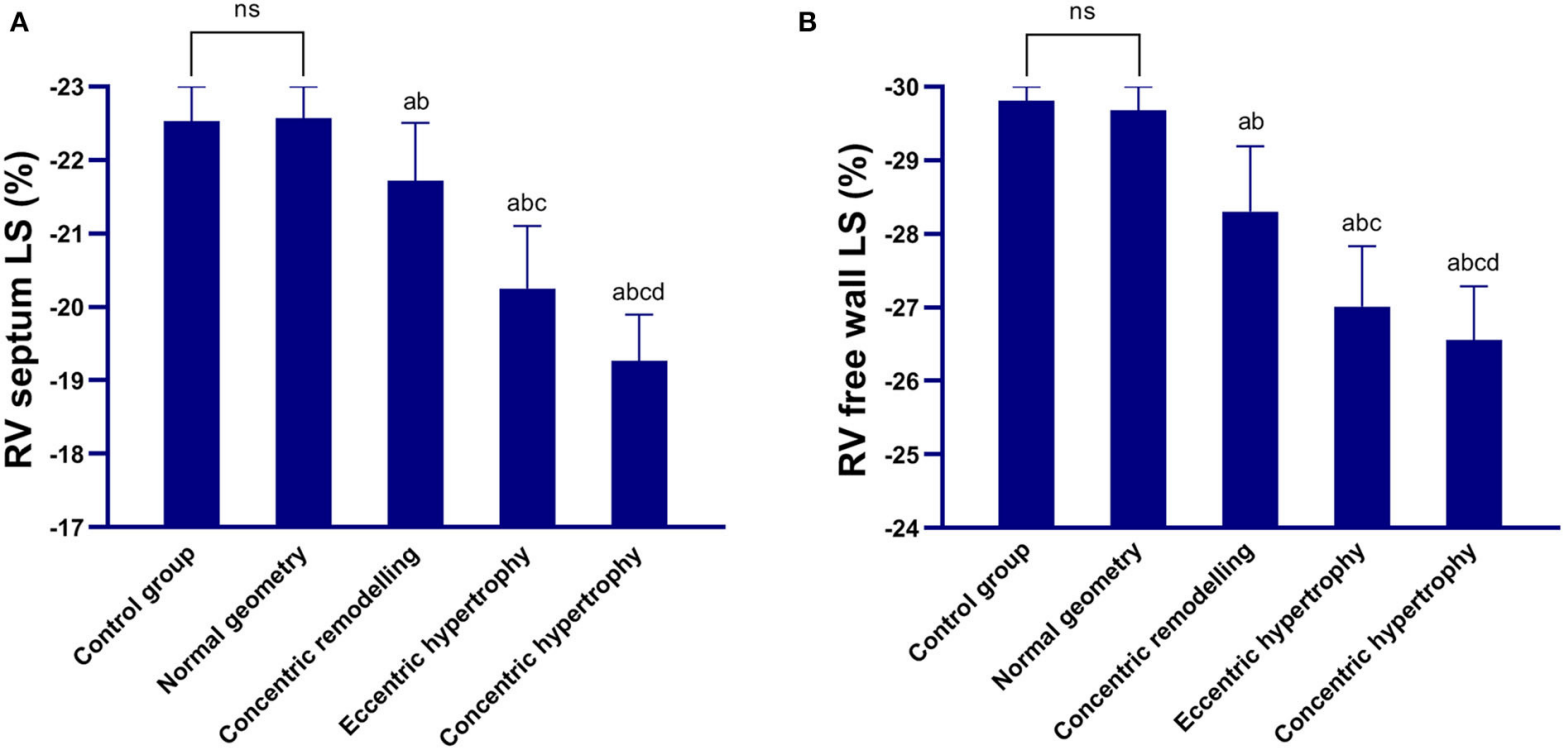
Comparison between the control group and four geometric pattern groups in terms of the longitudinal strain parameters of the right ventricular myocardium. **(A)** Right interventricular septum longitudinal strain; **(B)** Right ventricular free wall longitudinal strain. The right interventricular septum longitudinal strain and right ventricular free wall longitudinal strain decreased in the order of the normal geometric group, concentric remodeling group, eccentric hypertrophy group, and concentric hypertrophy group. ns: there was no significant difference between the control group and the normal geometric group; a: compared with the control group, *P* < 0.05; b: compared with the normal geometric group, *P* < 0.05; c: compared with the concentric remodeling group, *P* < 0.05; and d: compared with the eccentric hypertrophy group, *P* < 0.05.

The right ventricular free wall longitudinal strain and right interventricular septum longitudinal strain of the concentric remodeling, eccentric hypertrophy, and concentric hypertrophy groups were lower than those of the control and normal geometric groups, and the differences were statistically significant (*P* < 0.05). The right ventricular free wall longitudinal strain and right interventricular septum longitudinal strain were not significantly different between the normal geometric group and control group (*P* > 0.05) but significantly decreased in the order of the normal geometric group, concentric remodeling group, eccentric hypertrophy group, and concentric hypertrophy group (*P* < 0.05; Table 3 and Figure 1).

### Linear regression analysis

The univariate linear regression analysis suggested a statistically significant linear relationship between the SBP, hypertension duration, LVMI, RWT, RVEDV, and RVEF, and the right ventricular free wall longitudinal strain (*P* < 0.05). There was no statistically significant linear relationship between the other variables and the right ventricular free wall longitudinal strain (*P* > 0.05; Table 4).

**Table 4:** Univariate regression analysis results of right ventricular free wall longitudinal strain.

**Variate**	**Normalized β value**	***t* value**	***P* value**	**95%CI**
Gender	0.089	1.399	0.163	−0.110~0.647
Age	0.028	0.444	0.657	−0.054~0.085
SBP	0.419	7.245	< 0.001	0.100~0.174
DBP	0.084	1.328	0.186	−0.017~0.086
BMI	0.034	0.541	0.589	−0.224~0.394
Hypertension duration	0.289	4.739	< 0.001	0.066~0.159
HR	0.013	0.204	0.838	−0.031~0.038
IVSD	0.124	1.952	0.052	−0.001~0.320
LVPWD	0.104	1.647	0.101	−0.030~0.332
LVEDD	0.117	1.845	0.066	−0.004~0.131
LVESD	0.032	0.508	0.612	−0.052~0.089
LVEF	−0.111	−1.745	0.082	−0.178~0.011
LVMI	0.813	21.866	< 0.001	0.078~0.093
RWT	0.358	6.018	< 0.001	8.704~17.175
RVEDV	0.406	6.960	< 0.001	0.090~0.161
RVESV	0.057	0.900	0.369	−0.025~0.067
TAPSE	−0.117	−1.853	0.065	−0.195~0.006
FAC	−0.087	−1.369	0.172	−0.139~0.025
RVEF	−0.302	−4.977	< 0.001	−0.132~-0.057

The variables showing statistical significance based on the univariate linear regression analyses were included in the multivariable linear regression model for analysis. The results showed that the SBP, LVMI, and RWT were still statistically significant for the right ventricular free wall longitudinal strain after correcting other factors (*P* < 0.05). That is, those three factors were independently correlated with the right ventricular free wall longitudinal strain, and the LVMI had the dominant influence on the longitudinal strain (normalized β = 0.637, *P* < 0.05), followed by the SBP (normalized β = 0.202, *P* < 0.05) and RWT (normalized β = 0.155, *P* < 0.05; Table 5).

**Table 5:** Multivariate regression analysis results of right ventricular free wall longitudinal strain.

**Variate**	**Normalized β value**	***t* value**	***P* value**	**95%CI**
SBP	0.202	5.065	< 0.001	0.040~0.092
Hypertension duration	0.069	1.844	0.066	−0.002~0.055
LVMI	0.637	14.074	< 0.001	0.058~0.076
RWT	0.155	3.954	< 0.001	2.818~8.414
RVEDV	0.058	1.504	0.134	−0.006~0.041
RVEF	−0.045	−1.154	0.250	−0.038~0.010

### Repeated measures studies

**Figure 2:**
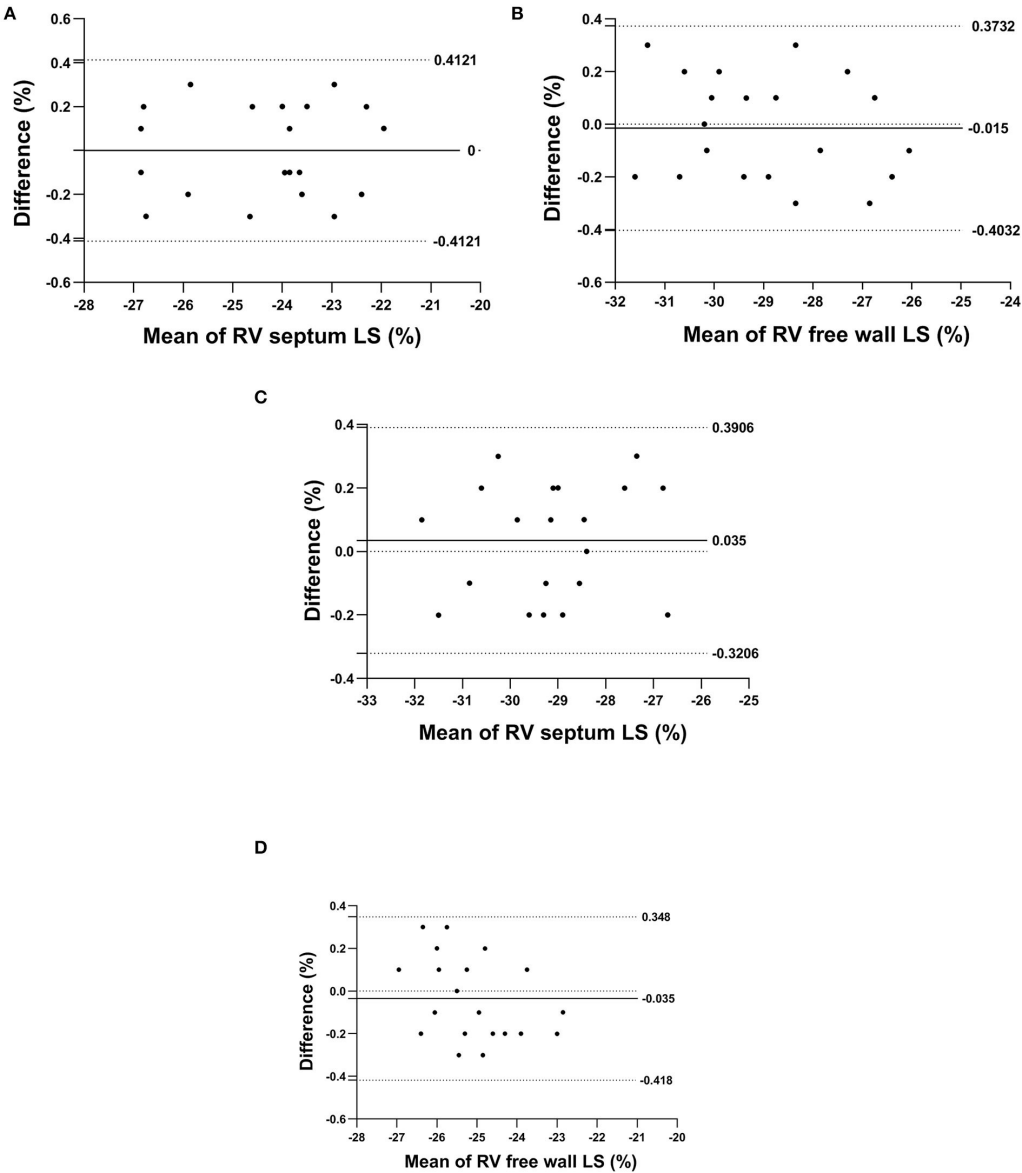
The Bland–Altman plot for repeated measurements of the right interventricular septum longitudinal strain and right ventricular free wall longitudinal strain; **(A,B)** denote the intra-observer agreement analyses, respectively, and **(C,D)** denote the inter-observer agreement analyses, respectively.

The same observer measured the right interventricular septum longitudinal strain and the right ventricular free wall longitudinal strain at different times and showed them to be 0.03 ± 0.80 and 0.05 ± 0.72, respectively. The right interventricular septum longitudinal strain and the right ventricular free wall longitudinal strain values measured repeatedly by different observers were 0.10 ± 0.68 and 0.07 ± 0.73, respectively. A Blant–Altman analysis showed that the repeatedly measured values changed in coherence with their average values (Figure 2).

## Discussion

The left ventricular remodeling resulting from hypertension is a complex multivariate process that can cause left ventricular morphological change to varying extents, leading to abnormal left ventricular geometric patterns and impaired cardiac function (14). Several studies have suggested that the pathophysiological factors causing left ventricular remodeling also affect the right ventricle; as the structures and functions of the bilateral circulatory systems are mutually restricted, the right ventricle is also affected during hypertension (15, 16). In this study, two-dimensional speckle-tracking imaging and three-dimensional echocardiography were used to evaluate the right ventricular deformation and function of elderly people with hypertension with different left ventricular geometric patterns. Significantly impaired right ventricular myocardial mechanics were found in those with abnormal left ventricular geometric patterns, especially concentric hypertrophy. The strain parameters of the concentric remodeling group with normal right ventricular volume and function parameters were significantly lower than those of the control and normal geometric groups. The right ventricular free wall longitudinal strain was independently correlated with the SBP, LVMI, and RWT.

In the case of long-term elevation of blood pressure, the fibroblast proliferation against the increasing left ventricular afterload leads to increased cardiomyocyte volume, resulting in an increased LVM and a thickened left ventricular wall (17). In this study, compared with the control group, the IVSTd, LVPWTd, and LVMI were increased in all abnormal geometric groups, especially the concentric hypertrophy group. The most remarkable increase in the LVEDD and LVESD was observed in the eccentric hypertrophy group. This may be ascribed to myocardial apoptosis or necrosis, breaking the cross-linking of collagen protein and increasing sarcomeres in extending myocardial fibers, meaning left ventricular chamber enlargement is a major characteristic of eccentric hypertrophy (18). Compared with the concentric hypertrophy group, the LVEF was increased in the control and normal geometric groups and was not remarkably decreased in the other geometric groups. An early geometric pattern change is an adaptive response to increased blood pressure. The left ventricular wall has a compensatory contraction to maintain the ejection fraction at a normal level. The left ventricular wall grows significantly thicker when the condition progresses to concentric hypertrophy, increasing stiffness and decreasing deformability. The ratio of blood vessels to myocardial fibers decreases, leading to a relatively insufficient blood supply to the myocardium. The oxygen metabolism imbalance leads to the decrease of LVEF (19).

In this study, the RVEDV and RVESV were increased in the left ventricular hypertrophy groups, especially the concentric hypertrophy group; this indicated that as the LVMI and left ventricular diameter increased, the structure of the right ventricular chamber changed in a similar manner (i.e., the right ventricular volume increased), consistent with previous studies (20). This was because the left ventricular end-diastolic volume of patients with left ventricular hypertrophy is increased, and pressure builds up and is transmitted posteriorly through the pulmonary circulation connecting two chambers, increasing the right ventricular afterload, affecting the right ventricular ejection function and leading to increased right ventricular volume. However, when the LVMI is normal, there is no remarkable change in the right ventricular volume.

Compared with the control and normal geometric groups, the RVEF and FAC were decreased in the eccentric hypertrophy and concentric hypertrophy groups, and TAPSE was decreased in the concentric hypertrophy group. This indicated that impaired right ventricular systolic function could be detected based on right ventricular function parameters in patients with hypertension with left ventricular hypertrophy. Hypertension may cause right ventricular dysfunction in the following way: First, overstimulation of the renin-angiotensin-aldosterone system and sympathetic nervous system produces vasoactive substances, oxidative stress, and endothelial dysfunction, which can induce accumulation of myocardial collagen and myocardial fibrosis, followed by remodeling of the left and right ventricles (20). At the same time, these changes lead to reactive contracture of the pulmonary artery and hypertrophy of the middle smooth muscle, increasing pulmonary circulation resistance, increasing right ventricular afterload, and promoting right ventricular remodeling (21). Secondly, due to the interdependence of the left and right ventricles, the left and right ventricles share the ventricular septum and the annular and spiral muscle fibers, which influence each other. When the left ventricular pressure and volume load increase, the right ventricular hemodynamics will also be affected (22, 23).

In patients with hypertension, as the LVMI increases, the continuous right ventricular myocardial proliferation and hypertrophy result in right ventricular myocardial fibrosis, wall thickening, decreased deformability, and progressive mechanical damage of the right ventricular myocardium (24). Tadic et al. (8) showed that the right ventricular myocardial mechanics of patients with left ventricular hypertrophy are significantly inferior to those without left ventricular hypertrophy. This study echoed Tadic et al. results. The right ventricular strain parameters decreased from the normal geometric group to the concentric hypertrophy group, and the latter showed the most significant damage.

The current study provides novel findings on the relationship between the right ventricular strain and the left ventricular geometric pattern. Compared with the concentric remodeling group, the right interventricular septum longitudinal strain and right ventricular free wall longitudinal strain increased in the control and normal geometric groups. That is, for the concentric remodeling group, the right ventricular longitudinal strain decreased when the volume and function parameters were still normal, indicating that the right ventricular myocardial mechanics of the patients with hypertension was impaired despite their normal TAPSE, FAC, and RVEF values. The right ventricular myocardium comprises subendocardial longitudinal muscle fibers and epicardial circumferential muscle fibers. Right ventricular ejection is primarily caused by contraction of the longitudinal muscle fibers, and the endocardium is more sensitive to pressure load and ischemia/hypoxia (25). Therefore, changes in the right ventricular systolic function are mainly reflected by decreased longitudinal function in prehypertension. The study results further demonstrated the sensitivity of the right ventricular longitudinal strain in detecting the right ventricular systolic function. Subclinical and subtle changes could be observed, e.g., decreased right ventricular myocardial mechanics in the early stage of hypertensive remodeling, consistent with the findings of previous studies (26).

As the interventricular septum is a component of the left ventricle, the right interventricular septum longitudinal strain may be affected by the left ventricular function; the right ventricular free wall longitudinal strain provides a more accurate estimation of the right ventricular myocardial function and prognostic information (27). Carluccio et al. (28) showed that the prognostic value of the right ventricular free wall longitudinal strain is better than that of the right interventricular septum in cardiovascular events. Therefore, this study examined the relationship between the right ventricular free wall longitudinal strain, clinical indexes, and related echocardiographic parameters in elderly patients with hypertension. According to the linear regression analysis results, the SBP, LVMI, and RWT are independently correlated with and have significant effects on the right ventricular free wall longitudinal strain; the LVMI has the dominant influence. That is, the damage risk for right ventricular free wall myocardial mechanics was found to increase with the LVMI in patients with hypertension, consistent with previous findings (8). This might be linked to the influence on the right ventricular systolic function *via* the helical muscle bundle, interventricular septum, and pericardium shared by the two ventricles, which increases with the LVMI (16). In addition, the SBP might affect the right ventricular free wall longitudinal strain as follows: when the blood pressure is elevated, various vasoactive substances mediated by activating the renin-angiotensin-aldosterone system could induce fibrosis and degeneration of the pulmonary vascular wall and intima, further affecting the right ventricular systolic function (29, 30). Soylu et al. (31) found a remarkably higher plasma aldosterone level in patients with concentric hypertrophy compared with patients with normal geometric and concentric remodeling, which might lead to different right ventricular myocardial mechanics for different left ventricular geometric patterns. Moreover, Todiere et al. (32) evaluated the effects of isolated hypertension on the left and right ventricular structures and functions by cardiac magnetic resonance, and the results showed a significant positive correlation between the LVM and left ventricular early peak filling rate. LVEF and the right ventricular mass, right ventricular early peak filling rate, and RVEF, respectively, indicate a certain correlation between hypertension-induced myocardial structural and functional abnormalities between the left and right ventricles. Finally, Tadic et al. (33) showed an independent correlation of the 24-h SBP and LVMI with the right ventricular free wall longitudinal strain, consistent with the findings of this study.

## Limitations

Aging is a risk factor for cardiovascular disease, so the impairment of cardiovascular function with age in elderly patients with hypertension could not be ruled out in this study. In addition, the cutoff values of the LVMI and RWT provided by this study were obtained from European and American populations. Normal reference values of echocardiography indexes in Chinese populations differ from those in Westerners, so these cutoff values should be further validated among Chinese patients. Finally, the patients' follow-up visits were not included in this study, and the improvement of the right ventricular myocardial mechanics was not analyzed *via* antihypertensive therapy. In this study, we randomly selected 20 patients for the reproducibility study, which may add an additional burden to patients.

## Conclusion

The right ventricular myocardial mechanics in the elderly with hypertension with abnormal left ventricular geometric patterns are significantly impaired, and the systolic function declines, especially in patients with concentric hypertrophy. In this study, compared with the right ventricular three-dimensional volume and function parameters, the right ventricular strain parameters could indicate mechanical damage in the concentric remodeling group earlier. The right ventricular free wall longitudinal strain was affected by the SBP, LVMI, and RWT (especially the LVMI). Therefore, clinicians should focus on the right ventricular function of patients with hypertension with abnormal left ventricular geometric patterns to identify subclinical right ventricular systolic dysfunction in the early stage, thus providing a basis for clinical treatment and prognosis.

## Data availability statement

The original contributions presented in the study are included in the article/supplementary material, further inquiries can be directed to the corresponding author.

## Ethics statement

The studies involving human participants were reviewed and approved by Ethics Committee of Shanxi Bethune Hospital. The patients/participants provided their written informed consent to participate in this study.

## Author contributions

CK, JX, and XK: conception and design of the research. JM, JX, and XK: acquisition of data. JM and QQ: analysis and interpretation of the data. XK and SL: statistical analysis. JX and QQ: writing of the manuscript. CK: critical revision of the manuscript for intellectual content. All authors read and approved the final draft.

## Conflict of interest

The authors declare that the research was conducted in the absence of any commercial or financial relationships that could be construed as a potential conflict of interest.

## Publisher's note

All claims expressed in this article are solely those of the authors and do not necessarily represent those of their affiliated organizations, or those of the publisher, the editors and the reviewers. Any product that may be evaluated in this article, or claim that may be made by its manufacturer, is not guaranteed or endorsed by the publisher.
